# Geometrically frustrated interactions drive structural complexity in amorphous calcium carbonate

**DOI:** 10.1038/s41557-023-01339-2

**Published:** 2023-09-25

**Authors:** Thomas C. Nicholas, Adam Edward Stones, Adam Patel, F. Marc Michel, Richard J. Reeder, Dirk G. A. L. Aarts, Volker L. Deringer, Andrew L. Goodwin

**Affiliations:** 1 Inorganic Chemistry Laboratory, Department of Chemistry, University of Oxford, Oxford, UK; 2 Physical and Theoretical Chemistry Laboratory, Department of Chemistry, University of Oxford, Oxford, UK; 3 Department of Geosciences, Virginia Tech, Blacksburg, VA USA; 4 Department of Geosciences, Stony Brook University, Stony Brook, NY USA

**Keywords:** Inorganic chemistry, Materials chemistry, Physical chemistry

## Abstract

Amorphous calcium carbonate is an important precursor for biomineralization in marine organisms. Key outstanding problems include understanding the structure of amorphous calcium carbonate and rationalizing its metastability as an amorphous phase. Here we report high-quality atomistic models of amorphous calcium carbonate generated using state-of-the-art interatomic potentials to help guide fits to X-ray total scattering data. Exploiting a recently developed inversion approach, we extract from these models the effective Ca⋯Ca interaction potential governing the structure. This potential contains minima at two competing distances, corresponding to the two different ways that carbonate ions bridge Ca^2+^-ion pairs. We reveal an unexpected mapping to the Lennard-Jones–Gauss model normally studied in the context of computational soft matter. The empirical model parameters for amorphous calcium carbonate take values known to promote structural complexity. We thus show that both the complex structure and its resilience to crystallization are actually encoded in the geometrically frustrated effective interactions between Ca^2+^ ions.

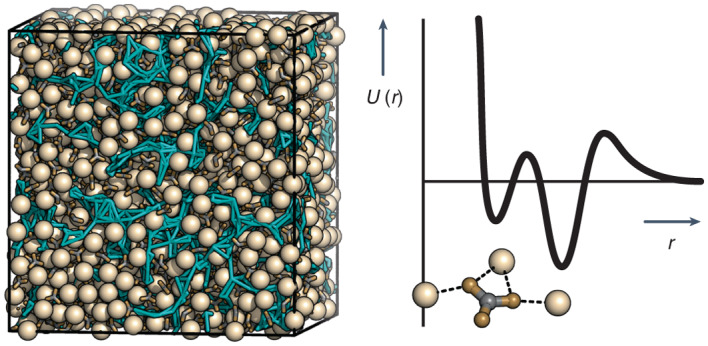

## Abstract (web summary)

Although amorphous calcium carbonate represents an important biomineralization precursor, its structure has been difficult to understand. Now, amorphous calcium carbonate’s structure is shown to arise from the different bridging modes available to the calcium ions. This effective multi-well potential that drives calcium arrangements creates a geometric incompatibility between preferred Ca–Ca distances and frustrates crystallization.

## Main

Calcium carbonate is relatively unusual among simple inorganic salts in that it precipitates from aqueous solution in a metastable hydrated, amorphous form^1^. Amorphous calcium carbonate (ACC)—with a nominal composition of CaCO_3_⋅*x*H_2_O (*x* ≈ 1)—can be stabilized for weeks by incorporating dopants such as Mg^2+^ or PO_4_^3−^ (refs. ^2,3^), or alternatively directed to crystallize into a number of different polymorphs by varying the pH or temperature^4^. Nature exploits this complex phase behaviour in a variety of biomineralization processes to control the development of shells and other skeletal structures^5,6^. Not only does the amorphous nature of biogenic ACC allow transformation to different crystalline CaCO_3_ polymorphs, but it helps organisms fashion larger-scale hierarchical morphologies that are important in biomineral architectures^7^. In seeking to develop bio-inspired crystal-engineering approaches for synthetic control over phase and morphology selection, there is an obvious need to understand why such a chemically simple system can exhibit such complex phase behaviour.

One domain in which a similar kind of phase complexity has been studied deeply from a theoretical perspective is that of soft-matter systems governed by multi-well pair potentials. Whereas isotropic particles that interact via a simple single-well potential (for example, Lennard-Jones, LJ) self-assemble into structurally simple crystalline phases (for example, face-centred cubic), the inclusion of one or more additional energy minima to the interaction potential can drive remarkable complexity if the distances at which these minima occur lead to geometric frustration^8–11^. An elegant example in two dimensions is that of quasicrystal self-assembly for specific parameters of the double-well Lennard-Jones–Gauss (LJG) potential^8,12^. In three dimensions, the same interaction model can be tuned to stabilize complex crystals with enormous unit cells^11^, and some combinations of the LJ and Gauss well positions even appear to frustrate crystallization altogether^9^.

That competing length scales might be relevant to ACC is a point hinted at by the observation of two preferred Ca⋯Ca distances dominating medium-range order in synthetic ACC^13,14^. These two distances are directly evident in the experimental X-ray pair distribution function (PDF) of ACC and are attributed to different bridging modes of the carbonate ion, which can connect a pair of Ca^2+^ ions either directly through one of the oxygen atoms (Ca–O–Ca pathway) or by inserting the carbonate ion fully between the cations (Ca–O–C–O–Ca pathway).

In this Article we explore the possibility that the structure of ACC is governed by effective interactions between Ca^2+^ ions that also reflect these two length scales, and that its well-known complexity emerges because the distances involved are in competition with one another. Our approach begins by obtaining a high-quality measure of the Ca-pair correlation function in ACC. We do this by applying a hybrid reverse Monte Carlo (HRMC) approach^15^ to generate the first structural model of ACC that is simultaneously consistent with experiment and stable with respect to state-of-the-art potentials. The Ca-pair correlation function that emerges is then inverted using a recently developed algorithm^16^ to reveal the effective, carbonate/water-mediated Ca⋯Ca interaction potential. We find that this potential is closely related to the LJG formalism, with empirical parameters that are known to frustrate crystallization. Monte Carlo (MC) simulations driven by our LJG model yield coarse-grained representations of ACC that capture key aspects of our fully atomistic HRMC models. In this way, we explain the structural complexity of ACC in terms of geometric frustration of two competing energetically favoured Ca⋯Ca separations. Mapping the problem of ACC structure onto the phase behaviour of multi-well potentials is important not only because it establishes the first experimental system for which these potentials are relevant, but also because it suggests how structural complexity in inorganic phases might be controllably targeted through suitable tuning of effective interactions.

## Results

### Structure of ACC

Our HRMC refinements made use of atomistic configurations containing 12,960 atoms (1,620 CaCO_3_⋅H_2_O formula units), with simulation cell sizes of ~5 nm. During refinement, atomic moves were proposed and then rejected or accepted according to a Metropolis Monte Carlo criterion, with a cost function that measured the quality of fit to X-ray total scattering data^17^ and also the energetic stability evaluated using the state-of-the-art interatomic potential of ref. ^18^. We favoured an HRMC approach over empirical potential structure refinement (EPSR), because calcium carbonate potentials are notoriously delicately balanced^18^, such that EPSR-derived potentials are unlikely to be physically meaningful for this system. To allow for direct comparison with previous studies, we used a weighted variant of the experimental X-ray total scattering function (that is, *QF*_X_(*Q*)) of synthetic ACC reported in ref. ^13^, which has been shown to be indistinguishable from that obtained for biogenic ACC samples^19^. For consistency, our HRMC configuration size and refinement constraints were also identical to those used in the reverse Monte Carlo (RMC) investigation of synthetic ACC in ref. ^14^. The absence of key energetic considerations in that RMC study allowed the development of physically unreasonable charge separation to give a model of cationic Ca^2+^-rich domains separated by channels of carbonate anions and water molecules. Our expectation was that the explicit consideration of electrostatics in our HRMC implementation would guide refinement towards a solution that was equally consistent with experiments, but also energetically sensible.

**Fig. 1:**
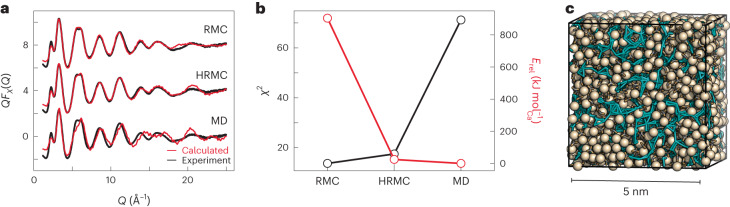
HRMC yields a balanced model of ACC structure. **a**, The experimental *Q*-weighted X-ray total scattering function *QF*_X_(*Q*) is well fitted by both RMC (top trace) and HRMC (middle) refinements, but is meaningfully different to that calculated using the interatomic potential of ref. ^18^ (bottom). Experimental data are shown as black lines, and fits or calculations as red lines. **b**, Quality criteria for the different structural models. HRMC simultaneously optimizes both goodness-of-fit to experimental data (*χ*^2^) and cohesive energy (*E*_rel_), and therefore finds a structure solution that is essentially as consistent with experiment as that obtained using RMC, while also as energetically sensible as that obtained using potentials alone (MD). **c**, Representation of a converged structure of ACC obtained using HRMC. Water molecules connect to form filamentary strands (aquamarine strings) that separate calcium carbonate-rich domains (Ca atoms are shown as large beige spheres, and carbonate ions as stick representation). Source data

**Extended Data Fig. 1:**
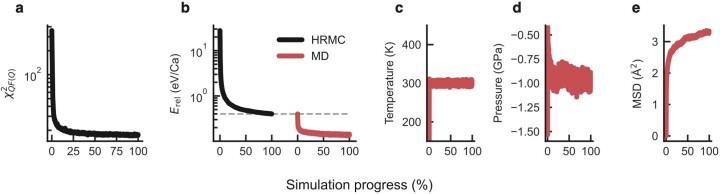
Evolution of system properties during HRMC refinement and MD simulation. **a** The cost function relating to the X-ray scattering data during HRMC refinement. **b** The change in potential energy of the simulated structural model during HRMC refinement (black) and subsequent MD simulation (red). Energies are reported relative to that obtained in equivalent *NVT* MD simulations of the crystalline monohydrocalcite polymorph of CaCO_3_.H_2_O. The **c** temperature, **d** pressure, and **e** mean-squared displacement (MSD) of atoms during the HRMC MD simulation, respectively.

HRMC does indeed find a suitable compromise between experiment and theory. We show in Fig. 1a the *QF*_X_(*Q*) function calculated from a representative configuration, and compare it to the equivalent functions predicted using pure RMC refinement, on the one hand, and unconstrained molecular dynamics (MD) simulations with the potentials of ref. ^18^, on the other hand. Both RMC and HRMC give very similar high-quality fits to experiment—unsurprising, of course, as they have been refined against these data. The MD simulation, however, misses some aspects of the *QF*_X_(*Q*) function. By contrast, the HRMC and MD models give similar cohesive energies, whereas the RMC model is less stable than both by more than 800 kJ mol^−1^ per formula unit. These results are summarized in Fig. 1b, which captures the motivation for our use of HRMC as a suitable balancing act: it has, for the first time, allowed access to an atomistic representation of ACC structure that is consistent with experiment and gives sensible energies using established hydrated calcium carbonate potentials. We also checked whether the HRMC model could reproduce the neutron total scattering data of refs. ^20–22^ (it can—see Supplementary Fig. 1 and Supplementary Discussion 1) and whether it is stable in MD simulations driven by the potentials of ref. ^18^ (it is—see Extended Data Fig. 1).

The HRMC model itself is illustrated in Fig. 1c. We observe that water is not homogeneously distributed throughout the configuration, but rather that the ACC structure consists of CaCO_3_-rich regions separated by a filamentary network of water: we term this a ‘blue cheese’ model. Qualitatively similar descriptions were obtained in MD simulations^23^, in EPSR refinements of combined neutron/X-ray total scattering data^22^, and also inferred from solid-state NMR measurements^24^. In all cases, the interpretation is that nearly all H_2_O molecules are bound to Ca^2+^ (as implied by ^1^H NMR; ref. ^25^), but neighbouring water molecules are sufficiently close to form a network that percolates the ACC structure. As anticipated, the charge separation that developed in the RMC model of ref. ^14^ has now vanished: the water-rich filaments we observe do not contain any free carbonate and are substantially narrower than the nanopore channels reported previously. The fact that RMC and HRMC models give similar *QF*_X_(*Q*) fits despite their very different descriptions of the structure reflects the difficulty of discriminating between C and O atoms using X-ray scattering methods alone^26^.

**Fig. 2:**
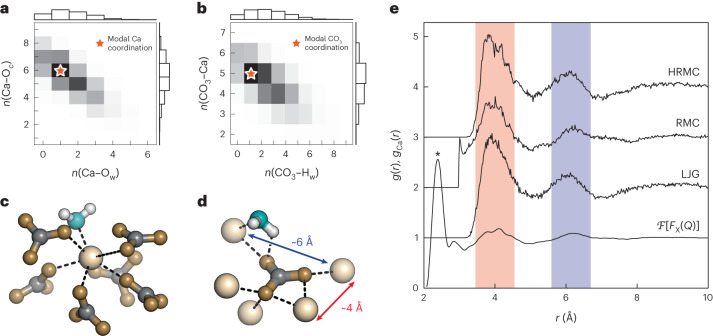
Coordination environments and Ca-pair distributions in ACC. **a**, Histogram of Ca^2+^ coordination environments, decomposed into contributions from carbonate and water oxygen donors (O_c_ and O_w_, respectively). **b**, Histogram of CO_3_^2−^ coordination environments, now decomposed into contributions from Ca^2+^ and water hydrogen donors (H_w_). **c**, Representative Ca^2+^ coordination sphere for the modal coordination environment marked by a star in **a**. **d**, Representative CO_3_^2−^ coordination sphere for the modal coordination environment marked by a star in **b**. Note that pairs of Ca^2+^ ions within the same CO_3_^2−^ coordination sphere either share a common oxygen donor (for example, red arrow) or are connected by Ca–O–C–O–Ca pathways (for example, blue arrow). Ca, C, O and H atoms are shown in beige, grey, aquamarine and white, respectively. **e**, Ca-pair correlation functions *g*_Ca_(*r*) extracted from HRMC, RMC and LJG configurations, compared against the normalized Fourier transform of the experimental X-ray total scattering function (which includes contributions from all atom pairs, for example, the Ca–O peak at 2.4 Å marked with an asterisk). The two principal peaks common to all functions, indicated by red and blue shading, can be assigned to the two types of Ca^2+^-ion pair illustrated in **d**. Source data

**Extended Data Fig. 2:**
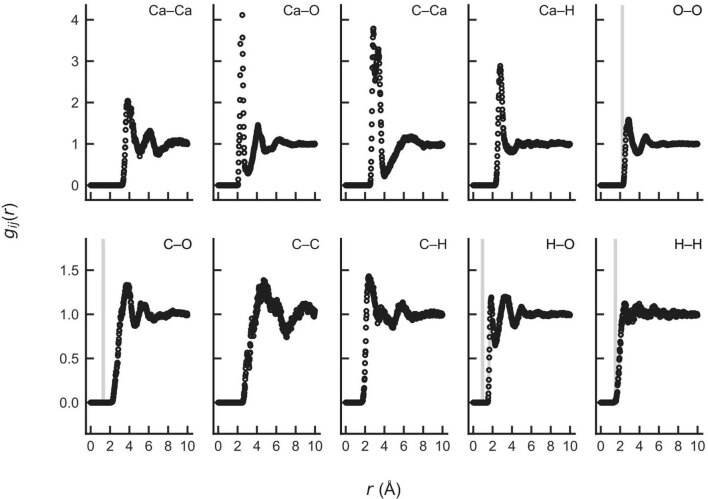
Partial pair distribution functions extracted from the HRMC ACC model. Each partial pair distribution function (PDF) is calculated with a bin width of Δ*r* = 0.02 Å, averaged over 12 HRMC trajectory configurations exported in intervals of 100,000 proposed moves. Sharp features arise from rigid-body correlations (light grey). For the relevant partial PDFs we omit rigid-body terms contributions from the data shown in black.

**Extended Data Fig. 3:**
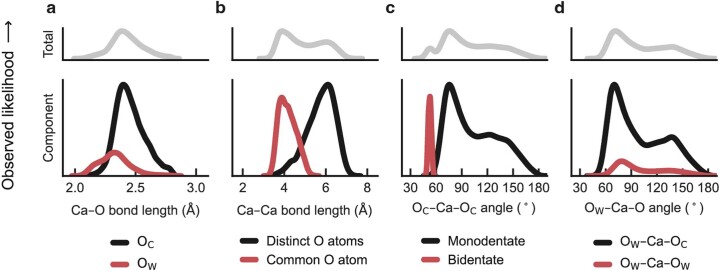
Key bond-length and bond-angle distributions in the HRMC ACC model. **a** The bond-length distributions for Ca-O divided into Ca-O_C_ and Ca-O_W_ contributions. The average Ca-O_C_ bond length (2.5(1) Å) is shifted to larger r values relative to the average Ca-O_W_ bond length (2.3(1) Å). **b** The interatomic distance between calcium atoms bound to the same carbonate can be divided into those which share a common oxygen atom and those which are connected via the carbonate molecule. These two coordination interactions give rise to the characteristic geometric frustration observed for ACC. **c** Bond-angle distributions for O_C_-Ca-O_C_ triplets, decomposed into mono- and bidentate coordination modes. The bidentate coordination mode gives rise to a sharp peak centred at 50^∘^. **d** O_W_-Ca-O bond-angle distributions. The lower relative intensity of O_W_-Ca-O_W_ correlations reflects the low likelihood of finding multiple water molecules bound to the same calcium atom. For each panel, the plots at the top show the corresponding cumulative distribution functions.

**Extended Data Fig. 4:**
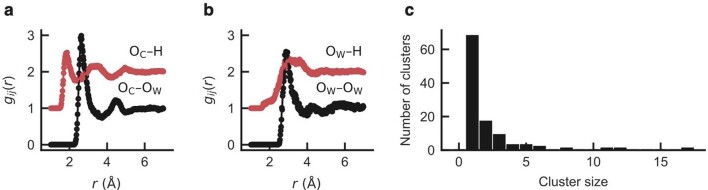
Characteristics of water distribution in the HRMC ACC model. **a** Radial distribution functions for neighbours of carbonate oxygen atoms in the structure. **b** Radial distribution functions for neighbours of oxygen atoms in water molecules (‘O_W_’) only. Intramolecular correlations are omitted for clarity. **c** The distribution of cluster sizes for those water molecules that do *not* take part in the percolating water network. A cluster size of 1 means an isolated water molecule that is not within reach of any others according to the defined cutoff distances (see Supplementary Table 1).

Locally, our HRMC model shows coordination environments that are consistent with the consensus of recent computational and experimental studies. For completeness, we show in Fig. 2a,b the distribution of calcium and carbonate coordination numbers. The average Ca^2+^ coordination number of 7.0—defined for a cutoff distance of 2.8 Å—is similar to that reported in refs. ^22,27,28^. Whereas most Ca^2+^ bind either five or six distinct carbonate anions, the carbonates tend to bind one fewer Ca^2+^ ion each, reflecting a binding-mode distribution of ~80:20 monodentate:bidentate. Representative modal coordination geometries for Ca^2+^ and CO_3_^2−^ are shown in Fig. 2c,d. As anticipated^14^, we find that carbonate anions bridge calcium-ion pairs in two ways: either a pair of Ca^2+^ ions share a common carbonate oxygen neighbour, or they bind distinct oxygens and so are connected formally by a Ca–O–C–O–Ca pathway. A full analysis of coordination environments, including bond-length and bond-angle distributions, is provided in Extended Data Figs. 1–4 and Supplementary Discussions 2 and 3.

### Coarse graining

In seeking to improve our understanding of the structure of ACC, we focused on the Ca-pair correlation function *g*_Ca_(*r*)—after all, this is the contribution to the pair distribution function that exhibits the strongest persistent well-defined oscillations. In Fig. 2e, we show this function as extracted from our HRMC configurations compared against that reported in the RMC study of ref. ^14^. We also include the normalized Fourier transform of the experimental *F*_X_(*Q*) function; this transform is an approximate (total) pair distribution function that emphasizes contributions from Ca–Ca pairs as a consequence of the larger X-ray scattering cross-section for Ca relative to C, O and H. Although all three real-space correlation functions show maxima at *r* ≈ 4 and 6 Å, it is the HRMC result that resolves these features most clearly. By counting Ca–Ca pairs separated by Ca–O–Ca and Ca–O–C–O–Ca pathways in our HRMC configuration, we confirmed that the two maxima in *g*_Ca_(*r*) occur at the preferred Ca⋯Ca distances associated with these two different carbonate bridging motifs (Extended Data Fig. 3). Indeed these same preferred separations recur among crystalline calcium carbonates, as noted in ref. ^14^.

**Fig. 3:**
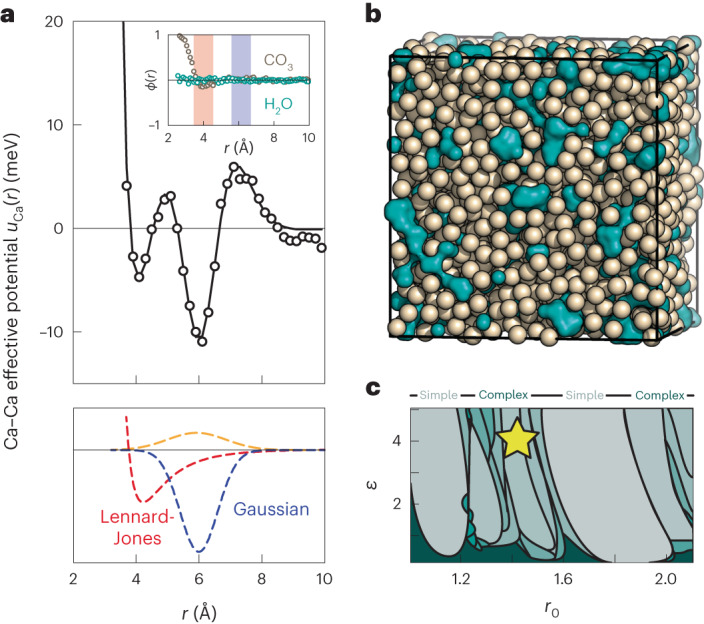
Effective Ca⋯Ca interactions in ACC. **a**, The effective Ca⋯Ca interatomic potential extracted from our HRMC configurations^16^ (open circles) and least-squares fit using a modified LJG model (line) as described in the text. The inset shows the orientational correlation functions (equation (1)), which vanish for distances relevant to the Ca⋯Ca separations. In the lower panel, the yellow dashed line corresponds to the additional broad, repulsive Gaussian term. **b**, Representative MC configuration of Ca atoms (beige spheres) generated by the LJG potential, parameterized by the fit shown in **a**. The Ca atoms are not uniformly distributed, but cluster to leave Ca-poor voids, shown as aquamarine surfaces. Note the qualitative similarity to the heterogeneous structure of ACC represented in Fig. 1c. **c**, Approximate location of ACC effective LJG parameters (star) in the LJG phase space as reported in ref. ^11^; shaded regions correspond to the stability fields of different ground-state structures, and solid lines denote the approximate locations of phase boundaries. Source data

Access to a smoothly varying measure of *g*_Ca_(*r*), together with the HRMC configurations from which it is calculated, allowed us to exploit a recently developed approach for the direct measurement of effective pair potentials from particle-coordinate data^16^. The method works by equating the pair distribution functions measured directly, on the one hand, and calculated using a test-particle insertion approach^29,30^, on the other^31^. Applying this methodology to the atomic coordinates in our HRMC-refined model, we extracted the effective Ca^2+^-ion pair potential *u*_Ca_(*r*) shown in Fig. 3a. This function has two distinct potential wells, the minima of which are centred near distances for which *g*_Ca_(*r*) has its maxima. The physical meaning of *u*_Ca_(*r*) is that it captures the effective two-body interactions between Ca–Ca pairs, mediated by carbonate and water, as required to account for the observed *g*_Ca_(*r*). That this interaction energy is minimized when Ca–Ca pairs are separated by distances corresponding to either Ca–O–Ca or Ca–O–C–O–Ca pathways makes intuitive sense (of course); the longer (6 Å) separation appears energetically more favourable, probably because it minimizes the electrostatic repulsion between calcium ions bound to a common carbonate.

One assumption in using an isotropic effective pair potential to capture interactions mediated by anisotropic molecular species, such as H_2_O and CO_3_^2−^, is that any local anisotropy is sufficiently short-ranged. We checked this point by calculating from our original (all-atom) HRMC configurations the orientational correlation functions:

1 $${\phi (r)}={\langle {P}_{2}({{{\bf{S}}}}\cdot {{{\bf{S}}}})\rangle }_{r}$$

where *P*_2_(*x*) is the second-order Legendre polynomial, and **S** are vectors parallel to a suitable local axis (for example, *C*_3_, *C*_2_) of molecules separated by distance *r* (ref. ^32^). We find that *ϕ*(*r*) is essentially featureless—for both carbonate and water—for all but the very shortest intermolecular approaches, which occur for distances smaller than the nearest-neighbour Ca⋯Ca separation (Fig. 3a, inset). Accordingly, the anisotropy of individual molecules is relevant only at a length scale smaller than that over which the effective interactions between Ca^2+^-ion pairs operate. The double-well form of *u*_Ca_(*r*) is important for a number of reasons. First, it explains why simple hard-sphere or LJ potentials fail to reproduce the experimental *g*_Ca_(*r*): if tuned to capture the 4 Å nearest-neighbour correlation, such potentials predict a second maximum just below 8 Å that is not observed in experiments. Second, it allows us to rationalize the inability of earlier EPSR studies to determine the correct Ca distribution function; for example, the study of ref. ^20^ included an LJ parameterization of Ca⋯Ca interactions that then resulted in a *g*_Ca_(*r*) function with maxima at positions clearly inconsistent with the inverse Fourier transform of the experimental *F*_X_(*Q*). Third, multi-well potentials are well known to support very complex phase behaviour^9,11,12,33–35^, suggesting a qualitative explanation of the complexity of ACC.

### LJG parameterization

To test this hypothesis of a link between effective potential and structural complexity, we sought to map *u*_Ca_(*r*) onto a suitable multi-well potential for which the corresponding theory is already well established. We found that a double-well LJG interaction^8^ described the observed functional form surprisingly well, as long as we included an additional broad, repulsive Gaussian term that helps capture the local maxima between and beyond the two minima of the LJG function. The behaviour of the LJG potential is characterized by three parameters—*ε*, *r*_0_ and *σ*—which describe, respectively, the depth, position and width of the Gaussian well relative to the LJ component^9,11^. A least-squares fit to *u*_Ca_(*r*) gave the values *ε* = 4.1, *r*_0_ = 1.4 and *σ* = 0.14 (Fig. 3a); note that *r*_0_ is particularly well defined because it is closely related to the ratio of the Ca⋯Ca separations resulting from the two different carbonate bridging motifs (~6 Å and 4 Å). We will return to the importance of these empirical parameters in due course. We note, in passing, related work on the description of coarse-grained molecular systems with double-well potentials, where one minimum is explicitly assigned to enthalpic and one to entropic terms^36^, and on the ‘learning’ of effective pair potentials from simulation data by back-propagation^37^.

**Extended Data Fig. 5:**
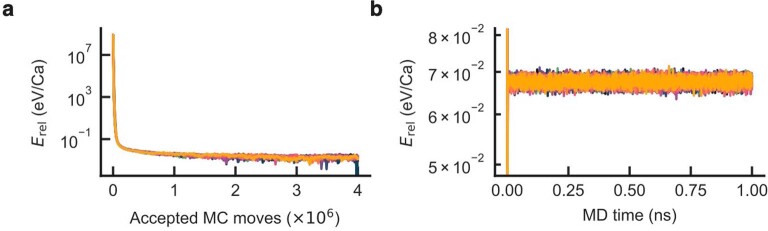
Evolution of coarse-grained simulation ensemble energies. The energy of each ensemble, relative to the minimum configuration energy across all trajectories, as a function of **a** accepted MC moves, and **b** MD simulation time. Each of the 12 trajectories is given a different colour. This initial sharp decrease and subsequent increase in *E*_rel_ for the MD configurations arises from the initially randomised particle positions, followed by a short energy minimisation, before beginning the *NVT* MD run.

As a simple check that our combination of *g*(*r*) inversion and potential parameter fitting does indeed result in a meaningful effective pair potential, we carried out direct MC simulations driven by our parameterized LJG potential. These simulations were performed using the experimentally determined Ca particle density in ACC, and although we focus here on MC simulations (for fairest comparison with HRMC), the same results emerge if we use MD instead (Methods and Extended Data Fig. 5). The corresponding pair correlation function closely matches our HRMC *g*_Ca_(*r*), as expected (Fig. 2e), and the resulting structure resembles the Ca distribution in the fully atomistic model (Fig. 3b; cf. Fig. 1c). But the similarity between coarse-grained LJG-driven and HRMC Ca distributions turns out to extend beyond pair correlations, and we explore this more extensive similarity in various forms (for example, ring statistics, Voronoi volume distributions and higher-order correlation functions) in Supplementary Figs. 2–4 and Supplementary Discussion 4. A particular point of interest is that the MC configurations contain Ca-rich/-poor regions that are qualitatively similar to those observed in the original atomistic model (Fig. 3b)—the clear implication being that, at the density of ACC, an inhomogeneous Ca distribution is encoded within the effective Ca⋯Ca interaction itself.

## Discussion

From the perspective of soft-matter theory, much of the interest in the LJG potential lies in the complexity of phase behaviour that it supports^9,11,12,38^. This complexity arises because of competition between the structure-directing effects of LJ and Gaussian components, which operate at different length scales. When the positions of the LJ and Gaussian minima are related by a factor 1.2 ≤ *r*_0_ ≤ 1.6, there is particularly strong geometric frustration that results in a general resistance to crystallization^9^ and the emergence of many competing low-symmetry ground-state structures^11^. This behaviour extends across a wide variety of relative well depths *ε* ≥ 1 for the corresponding relative Gaussian width *σ* = 0.14 (ref. ^11^). Hence the empirical parameters we have determined to be relevant to effective Ca⋯Ca interactions in ACC—that is, *r*_0_ = 1.4, *ε* = 4.1 and *σ* = 0.14—locate the system in one of the most complicated parts of the LJG phase diagram (Fig. 3c)^11^. In developing this mapping onto the LJG model, we assume that the additional broad Gaussian term we have included in our parameterization does not strongly influence the phase behaviour (note that the value of *r*_0_ does not vary substantially if it is omitted from our fit). Similarly, we cannot be certain of the effects of finite temperature and fixed density on LJG phase behaviour, as these have not yet been studied from theory. It is nevertheless a general phenomenon in frustrated systems that regions of strong geometric frustration are characterized by the existence of many competing ground states with suppressed ordering temperatures, above which the system is disordered but far from random^39,40^.

In the context of ACC, the implications are twofold. First, the different favoured Ca⋯Ca separations for carbonate-bridged Ca pairs—that is, 4 and 6 Å—are configurationally difficult to satisfy in a three-dimensionally periodic (that is, crystalline) structure with the density of ACC and in the absence of anisotropic interactions. This rationalizes, in general terms, why an amorphous form of hydrated CaCO_3_ is so readily accessible. Second, the sensitivity of the LJG potential around *r*_0_ = 1.4 hints—again in general terms—at why ACC might be directed to crystallize into a number of different polymorphs. Of course, as the water content of ACC varies during ageing of the material, both the effective Ca⋯Ca interactions and the bulk density must change, which makes extrapolation from the LJG effective potential of ACC difficult. Increased material density also strengthens the orientational correlations in molecular components, and so one expects the isotropic effective pair potential description to break down as crystallization is approached. Nevertheless, at the density of ACC and in the absence of orientational order, the occurrence of an amorphous state is now more easily rationalized. Furthermore, the incorporation of Mg^2+^ or PO_4_^3−^ ions into ACC will introduce statistical variations into the effective potential governing cation arrangements, favouring further the amorphous state—much as magnetic exchange disorder can stabilize spin glasses^41^.

Whereas the (hitherto unsuccessful) search for ‘real-world’ materials governed by isotropic multi-well potentials has focused on the atomic (alloys) and mesoscopic (colloids) length scales^11,12^, our study of ACC suggests that the intermediate domain of nanoscale materials may in fact be a more fertile source of relevant examples. By their very nature, molecular ions tend to exhibit a range of coordination modes, which must give rise to multiple specific preferred distances between the counterions they coordinate. Whenever these distances are geometrically frustrated, as in ACC, one expects that the corresponding effective potential must contain multiple wells so as to stabilize both separations at once. Through suitable choice of chemical components, one might hope to navigate the complicated phase space associated with such unconventional potentials. Doing so will provide an important test of theory, on the one hand, and also allow the targeted design of complex structures through self-assembly, on the other.

Our coarse-grained approach to understanding ACC structure may be applicable to other poorly ordered inorganic solids of particular scientific importance. Amorphous calcium phosphate (the precursor to bone) and calcium–silicate–hydrates (that is, Portland cements) are obvious ever-contentious examples^42,43^ where one might expect the different bridging motifs—now of the phosphate or (poly)silicate anions—to again moderate an effective interaction potential with multiple minima. Better understanding the structures of long-studied materials is certainly one fruitful avenue for future research, but our work also suggests an alternative pathway towards complex materials design. By varying the inorganic cation radius for a given molecular counterion, or by changing the degree of hydration, one might hope to control the ratio of the distances at which minima in the effective potential occur. What emerges is a strategy of ‘interaction engineering’ to target a particular phase of interest, or to stabilize or destabilize an amorphous form of matter.

## Methods

### HRMC refinements

Our approach to carrying out HRMC refinements was modelled closely on the previous RMC refinements of ref. ^14^, which were carried out using the RMCProfile code^44^. For the present study we used a related custom code, written in Python. The X-ray scattering calculations carried out by this code were exactly those employed by RMCProfile, but the code was also able to interface with potential-energy evaluations carried out using the LAMMPS software^45^. The custom Python code imports functionality from the following packages: PyLammpsMPI^46^, Numba^47^ and ASE^48^.

Starting configurations were generated in the following manner. We began by placing 1,620 Ca atoms randomly in a box of dimensions 52.8 × 54.8 × 45.2 Å^3^—this being the same box size as used in ref. ^14^. An initial energy minimization was carried out with a repulsive potential between Ca atoms (an LJ functional form, truncated at the zero-crossing distance), so as to prevent any Ca–Ca distances of <3.0 Å. To this homogeneous distribution of Ca atoms we added a stoichiometric number of rigid carbonate and water molecules with idealized geometries. Carbonate molecules were fixed to adopt a C–O bond length of *r*_CO_ = 1.284 Å—as observed in calcite and as defined for the relevant CaCO_3_ potential^18^. Water molecules were treated using the four-site rigid model, TIP4P-Ew^49^, which uses a fixed geometry of the water molecule (O–H bond length *r*_OH_ = 0.9572 Å, H–O–H bond angle *θ*_HOH_ = 104.52°). Short-range repulsive terms (as described above) were applied between the rigid bodies to prevent close contacts during a subsequent energy minimization and a short *NVT*-ensemble MD run, aiming to achieve a homogeneous distribution of particles (cf. the method of ref. ^50^).

The HRMC cost function, to be optimized during refinement, was defined as

2 $${\chi }^{2}={\chi }_{QF(Q)}^{2}+\frac{E}{{k}_{{{{\rm{B}}}}}T}$$

where

3 $${\chi }_{QF(Q)}^{2}=\mathop{\sum}\limits_{j}\frac{{\left[{Q}_{j}{F}_{{{{\rm{calc}}}}}({Q}_{j})-{Q}_{j}{F}_{{{{\rm{expt}}}}}({Q}_{j})\right]}^{2}}{{\sigma }^{2}}$$

represents the mismatch between calculated and experimental *Q*-weighted X-ray total scattering functions *QF*(*Q*) (ref. ^51^), and *E* is the configurational energy as obtained from the empirical potential. The experimental *QF*(*Q*) data were those reported in ref. ^13^ and ref. ^14^, which span the range of momentum transfers 1.2 ≤ *Q* ≤ 25.0 Å^−1^. The corresponding HRMC *QF*(*Q*) function was computed by weighted Fourier transform of the partial pair correlation functions, *g*_*ij*_(*r*) (refs. ^44,51^), themselves obtained as histograms with a bin width of Δ*r* = 0.02 Å. Total-scattering calculations were parallelized using the package Numba, a just-in-time compiler for Python^47^. The value of *E* was determined using the rigid-body force field for aqueous calcium carbonate of ref. ^18^, with the corresponding calculation delegated to the LAMMPS software^45^ via the PyLammpsMPI parallel LAMMPS–Python interface^46^. The simulation temperature was set to *T* = 300 K.

The parameter *σ* in equation (3) controls the relative weights of fit-to-data and potential energies in the overall HRMC cost function, and its value must be determined empirically. Through systematic tests of the variation in ${\chi }_{QF(Q)}^{2}$ and *E* with *σ*, we determined the value *σ* = 0.057 to provide the most appropriate balance in this case.

Individual moves were proposed and accepted or rejected according to the usual Metropolis–Hastings criterion. These moves involved a combination of atomic displacements (maximum value 0.1 Å) and rigid-body translations or rotations (maximum values of 0.1 Å and 1°, respectively) of carbonate and water molecules. In contrast to the RMC study of ref. ^14^, no closest-approach constraints were employed. The incorporation of interatomic potentials within the HRMC cost function ensured that such constraints were unnecessary.

HRMC refinements were continued until the values of *χ*^2^ and *E* were deemed to have converged (Extended Data Fig. 1).

### MD simulations

MD simulations were performed using LAMMPS^45^ in the *NVT* ensemble at 300 K, with a Nosé–Hoover thermostat controlling the temperature. The time step was 1 fs with a relaxation time of 0.1 ps for temperature control (cf. the ACC MD simulations in refs. ^27,52^). We tested the stability of our HRMC ACC structural model by carrying out a 1-ns MD simulation (one million time steps) starting from the final HRMC configuration. The simulation was stable with respect to *NVT* MD (Extended Data Fig. 1), by which we mean that no substantial structural reorganization occurred.

As anticipated, the HRMC configuration is indeed a compromise between fit-to-data and energy minimization. During the MD simulation, it quickly decreased in energy by ~0.3 eV per Ca before reaching thermal equilibrium (Extended Data Fig. 1). The negative pressure (Extended Data Fig. 1) indicates that the system is driven towards a more compact atomic arrangement than implied by the experimental density. An MD simulation performed in the *NPT* ensemble led to an increase in density from 2.43 to 2.91 g cm^−3^.

### Pair distribution function inversion

The test-particle pair distribution function inversion method was carried out using the code described in ref. ^30^, modified for application to a three-dimensional system. The Ca coordinates and the averaged *g*_Ca_(*r*) (up to a cutoff of 12 Å with a bin size of Δ*r* = 0.2 Å) from the final 12 frames of the HRMC trajectory were used as input for the inversion algorithm. Approximately 10,000 trial insertions were used per configuration, so as to satisfy convergence criteria.

### Coarse-grained simulations

MC and *NVT* MD simulations were performed using a custom Python program with potential-energy evaluations and MD protocols carried out using the LAMMPS software^45^.

Twelve starting configurations for each method (24 in total) were generated by randomly placing 1,620 Ca atoms in a box with identical cell dimensions as the HRMC configuration (52.8 × 54.8 × 45.2 Å^3^). The simulation temperature was set to *T* = 300 K (cf. HRMC simulations). For MC simulations, the maximum atomic displacement was 0.1 Å. MC simulations were terminated after four million moves were accepted, at which point convergence with respect to the ensemble energy was met (Extended Data Fig. 5). An initial energy minimization (using the the conjugate-gradient algorithm for 100 steps) was performed for the starting MD configurations to prevent close contacts rendering the simulation unstable. This gave an average minimum energy of −0.03028 ± 0.00090 eV per Ca. The MD simulations were then run in the *NVT* ensemble for 1 ns, with a 0.5-fs time step and 50-fs temperature damping constant.

## Online content

Any methods, additional references, Nature Portfolio reporting summaries, source data, extended data, supplementary information, acknowledgements, peer review information; details of author contributions and competing interests; and statements of data and code availability are available at 10.1038/s41557-023-01339-2.

### Supplementary information

Supplementary Information Supplementary Figs. 1–4, Table 1, Discussions 1–4 and references.

Supplementary Data 1 Atomic coordinates in crystallographic information file format for a representative hybrid reverse Monte Carlo model of amorphous calcium carbonate.

Supplementary Data 2 Atomic coordinates in crystallographic information file format for a representative reverse Monte Carlo model of amorphous calcium carbonate.

Supplementary Data 3 Atomic coordinates in crystallographic information file format for a representative Molecular Dynamics model of amorphous calcium carbonate.

Supplementary Data 4 Atomic coordinates in crystallographic information file format for a representative coarse-grained Lennard-Jones–Gauss model of calcium arrangements in amorphous calcium carbonate.

Supplementary Code 1 Python scripts used to carry out HRMC refinements as discussed in the main text.

### Source data

Source Data Fig. 1 Statistical source data.

Source Data Fig. 2 Statistical source data.

Source Data Fig. 3 Statistical source data.

## Data Availability

Data supporting the findings of this study are available within the paper and its Supplementary Information files, or at 10.5281/zenodo.8238547. Source data are provided with this paper.

## Code availability

All custom code used in this study was developed using widely available algorithms. A copy of the Python scripts used to carry out the HRMC refinements is included as Supplementary Code 1.
