# Supplementary material for: Geometrically frustrated interactions drive structural complexity in amorphous calcium carbonate
Source: Nat Chem. 2023 Sep 25;16(1):36–41. doi: 10.1038/s41557-023-01339-2 (PMC10774122; doi:10.1038/s41557-023-01339-2)
Supplement: Supplementary file 1 — Supplementary Figs. 1–4, Table 1, Discussions 1–4 and references. [file 41557_2023_1339_MOESM1_ESM.pdf]

nature chemistry

Article https://doi.org/10.1038/s41557-023-01339-2

Geometrically frustrated interactions drive structural complexity in amorphous calcium carbonate

In the format provided by the authors and unedited

Contents

Supplementary Figures 2

Supplementary Figure 1 2

Supplementary Figure 2 3

Supplementary Figure 3 3

Supplementary Figure 4 4

Supplementary Table 5

Supplementary Table 1 5

Supplementary Discussions 6

Supplementary Discussion 1. Comparison with neutron total scattering measurements 6

Supplementary Discussion 2. Detailed analysis of the HRMC ACC configuration . . . 7

Supplementary Discussion 3. The role of water in ACC 9

Supplementary Discussion 4. Comparing calcium-ion distributions in HRMC and LJG configurations 10

Supplementary References 12

Supplementary Figures

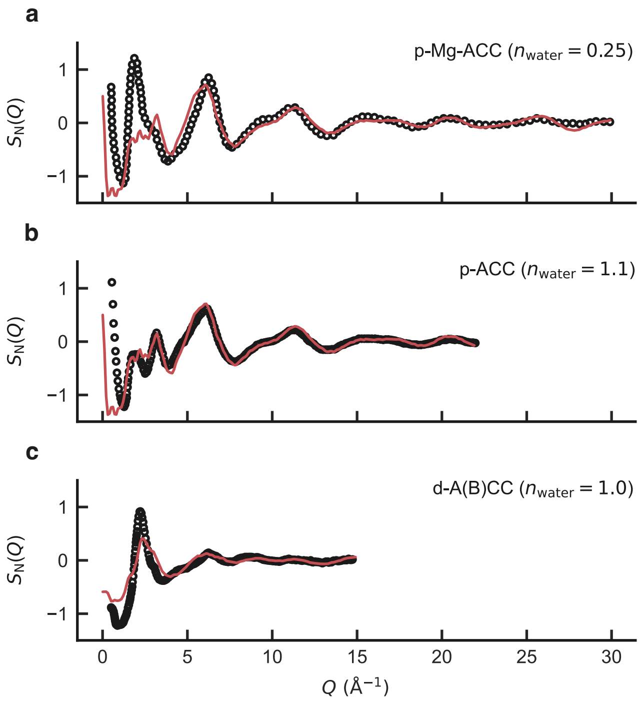

Supplementary Figure 1. Neutron total scattering function $S_N(Q)$ for the HRMC model compared to various experimental measurements. a $S_N(Q)$ for magnesium stabilised ACC (experimental data from Ref. 20). The sample was reported with a Mg:Ca ratio of 0.05:1, 0.25 H_2O molecules per Ca ("n_{water}"), and a mass density of 2.19 g/cm^3. The sample was intentionally dehydrated to reduce the effects of inelastic and incoherent scattering from protons. **b** $S_N(Q)$ for ACC with 1.1 H_2O molecules per Ca and mass density of 2.28 g/cm^3 (experimental data from Ref. 22). **c** $S_N(Q)$ for deuterated amorphous (basic) calcium carbonate, d-A(B)CC (experimental data from Ref. 21). The sample was reported to contain ≈ 10 % H/D exchange—which was accounted for in our calculation by choosing the appropriate ratio of the neutron scattering lengths for H/D.

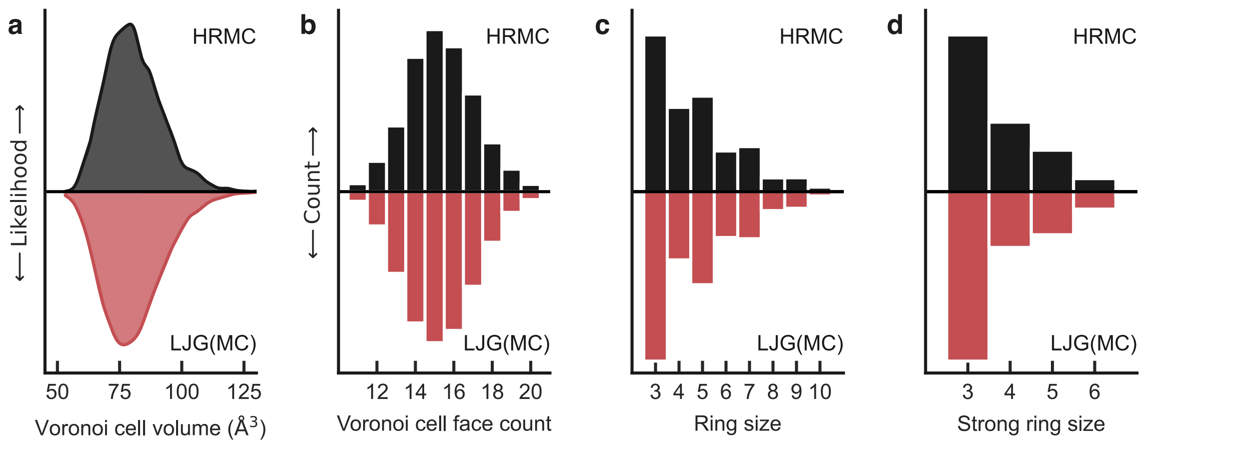

Supplementary Figure 2. Comparing local-structure properties in the HRMC (black) and Monte Carlo-driven LJG (red) configurations. The **a** Voronoi volume and **b** cell face count distributions in both configurations are similar. The distribution of **c** ordinary ring, and **d** strong ring sizes.

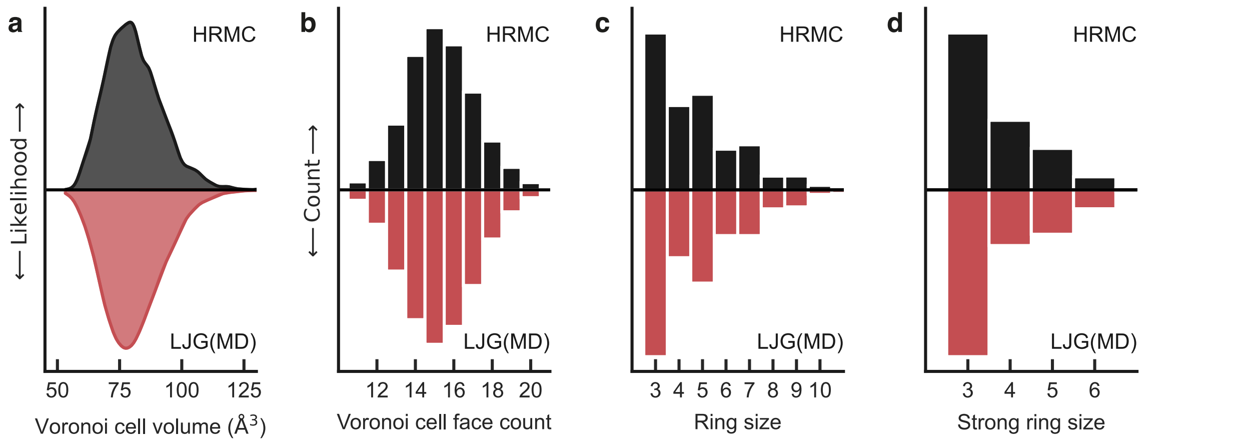

Supplementary Figure 3. Comparing local-structure properties in the HRMC (black) and molecular dynamics-driven LJG (red) configurations. The **a** Voronoi volume and **b** cell face count distributions in both configurations are similar. The distribution of **c** ordinary ring, and **d** strong ring sizes.

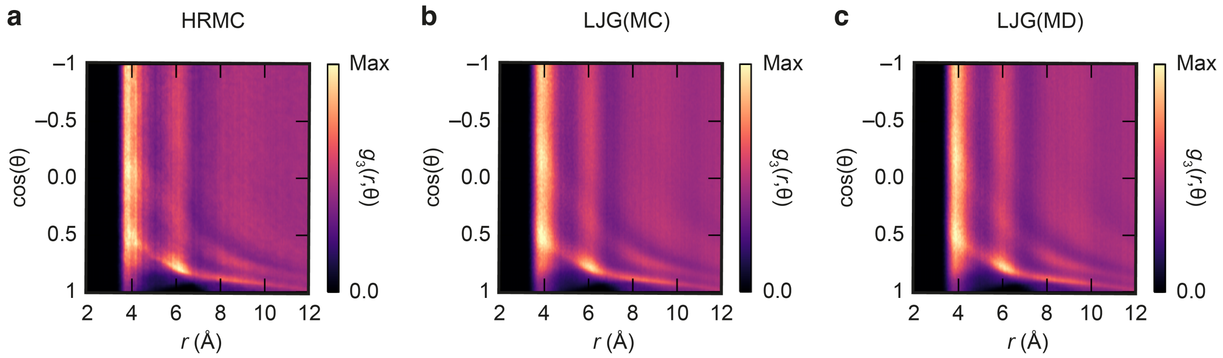

Supplementary Figure 4. Ca-triplet three-body correlations. The three-body correlation function $g_3(r, \theta)$ extracted from **a** HRMC, **b** LJG(MC), and **c** LJG(MD) configurations.

Supplementary Table

Supplementary Table 1. Radial cut-offs for determining local coordination environments.

Species	Atoms involved	Cut-off (Å)
Ca–CO_3	Ca, O_C	2.80
Ca–H_2O	Ca, O_W	2.80
CO_3–H_2O	O_C, H	2.30
H_2O–H_2O	O_W, O_W	3.75

Supplementary Discussions

Supplementary Discussion 1. Comparison with neutron total scattering measurements

It is notoriously difficult to obtain from neutron total scattering measurements of ACC a measure of the structure factor on an absolute scale. This limitation is a consequence of a number of factors: the mismatch in ACC stabilisation and neutron total scattering measurement timescales; facile H/D exchange, which leads to a large incoherent scattering background; and variation in $(H/D)_2O$ content during measurement. We are aware of three independent neutron total scattering measurements of ACC, all of which give qualitatively different total scattering functions [Supplementary Figure 1];[20–22] indeed this discrepancy is the reason for our focus here on X-ray total scattering data.

We show in Supplementary Figure 1 the best match between the neutron total scattering function calculated from our HRMC configuration and the three experimental data sets of Refs. 20–22. In calculating the corresponding neutron total scattering functions, we have varied the H/D ratio and $(H/D)_2O$ content in order to obtain the best match to data—noting that this is a key contributor to the experimental uncertainties involved in the measurements themselves.

Supplementary Discussion 2. Detailed analysis of the HRMC ACC configuration

In this section, we provide additional structural analysis of the main structural model of ACC reported in the present work.

Partial pair distribution functions

The partial pair distribution functions, $g_{ij}(r)$, as extracted from our HRMC configurations are shown in Extended Data Figure 2. For $g_{ij}(r)$ containing atoms associated with rigid-body constraints (*e.g.* intramolecular C–O bonds), sharp features are observed (light grey). For clarity, we represent the functions without intramolecular correlations (black dots), instead focusing on the atom pairs that are allowed to move freely in the HRMC refinement process.

Coordination statistics

Coordination statistics were evaluated for the final HRMC configuration using the bond-length cut-offs listed in Supplementary Table 1. Calcium is, on average, bound to 7.0(8) oxygen atoms, of which 5.5(13) (1.5(11)) correspond to carbonate (water) molecules, respectively. Calcium is bound to 4.5(10) carbonate molecules on average. The carbonate–calcium coordination shows a 79:21 monodentate:bidentate density distribution. This distribution agrees with the large difference in the average ^{13}C chemical shift between ACC and the crystalline polymorphs with bidentate carbonate binding motifs (aragonite and monohydrocalcite).[13] There are no calcium atoms without any bound carbonate molecules. On the other hand, about 20% of calcium atoms do not have any water molecules in their coordination sphere.

Carbonate molecules contain 2.2(15) water molecules in their coordination sphere, where a bonding interaction is defined by having a hydrogen bond between a water proton and carbonate oxygen. About 12% of carbonate molecules do not contain any water molecules in their coordination sphere.

The water molecules are found predominantly in filaments running through the $CaCO_3$ framework, with a preference for binding to Ca^{2+} over binding other water molecules. About 46% (53%) of water molecules are found to coordinate to one (two) calcium atoms, while only about 1% of water molecules are not bound to any Ca atoms (according to the strict cut-off definition provided in Supplementary Table 1). A very small number—7 of 1620 water molecules—

are shared amongst the coordination sphere of three calcium atoms each.

The coordination statistics are consistent with a structure dominated by a calcium-carbonate framework decorated with small water channels.

Key bond-length and bond-angle distributions

We show in panels (a) and (b) of Extended Data Figure 3 the contributions to the Ca–O partial pair distribution function arising from nearest-neighbour Ca–O_W and Ca–O_C bonds.

The assertion is made in the main text that the 4 and 6 Å peaks in the Ca–Ca partial pair distribution function correspond to Ca^{2+}-ion pairs connected by, respectively, a common O atom and distinct O atoms of a common carbonate molecule. This point is substantiated by the Ca–Ca bond-length distributions shown in panel (b) of Extended Data Figure 3.

In panels (c) and (d) of Extended Data Figure 3 we illustrate the bond-angle distributions associated with key O–Ca–O triplets. Panel (c) shows the distribution of O_C–Ca–O_C angles, decomposed into their contributions from bidentate and monodentate carbonate ligands. The former give rise to a sharp peak centred near 50°, whereas the latter correspond to a broad range of bond angles spanning 50–180°. Panel (d) shows the contributions arising from O_W–Ca–O_C and O_W–Ca–O_W triplets; these contributions are relatively similar in distribution to one another. The lower relative intensity for the O_W–Ca–O_W correlation compared to the O_W–Ca–O_C correlation reflects the small number of calcium environments bound by two (or more) water molecules.

Supplementary Discussion 3. The role of water in ACC

The emerging consensus on the role of water in the ACC is that water is coordinated predominantly by calcium and carbonate ions rather than forming any extended liquid-like structure dominated by water–water interactions. This point has been flagged in the analysis of both MD simulations[50] and neutron total scattering experiments.[20–22,53] Given the reasonable match of our HRMC configuration to the computed neutron scattering functions [Supplementary Figure 1], and the fact that the interatomic potential enables an explicit modelling of the energetics of the $Ca\cdots H_2O$, $CO_3\cdots H_2O$, and $H_2O\cdots H_2O$ interactions, the HRMC configuration also confirms the previously established existence of network-like water filaments throughout a $CaCO_3$ matrix.

Inspecting the radial distribution functions relevant to the water-based interactions, we note the presence of a peak at ≈ 1.8 Å for the $g_{\mathrm{O_CH}}(r)$ function, and its absence in the corresponding $g_{\mathrm{O_WH}}(r)$ function [Extended Data Figure 4, panels (a) and (b), respectively]. This observation indicates a preferential H-bonding interaction between water and carbonate molecules, rather than between water molecules, which can be rationalised in terms of an energetic preference for electrostatic interactions.

The application of percolation theory to MD-derived atomistic models of ACC has suggested that the percolation transition of water through the structure occurs at a hydration level of $x \approx 0.8$ for $CaCO_3 \cdot xH_2O$.[50] Indeed, our HRMC model ($x = 1.0$) exhibits percolating water channels with 1404 (of 1620) water molecules participating in a percolating cluster. In this calculation, we have assumed that water molecules are connected when $r_{\mathrm{O_WO_W}} \leq 3.80$ Å, which is the same upper limit as that used in Ref. 50. The remaining ("finite-size cluster") water molecules are predominantly found as lone species, however non-percolating clusters containing as many as 17 water molecules are found in our HRMC configuration [Extended Data Figure 4, panel (d)].

Supplementary Discussion 4. Comparing calcium-ion distributions in HRMC and LJG configurations

To confirm that the effective Ca$\cdots$Ca interaction potential was capable of capturing structural characteristics beyond the $g_{\mathrm{Ca}}(r)$ correlations alone, we compared the distribution of Voronoi cell volumes and cell face counts, ring statistics, and a mixed radial/angular three-body distribution function, $g_3(r,\theta)$,[54] for the HRMC Ca-only and the LJG configurations [Supplementary Figures 2–4]. We show the property distributions comparing HRMC with both the MC- and MD-driven LJG configurations—denoted LJG(MC) and LJG(MD), respectively—as evidence that the results are independent of the choice between MC and MD methods. Voronoi properties were computed using the `freud` library.[55] Ring statistics were computed using the `PeriodicGraphs` Julia package.[56] The $g_3(r,\theta)$ correlation functions were computed using the code from Ref. 54. HRMC properties were averaged over the final 12 trajectory snapshots. All LJG properties were computed as ensemble averages from the final frames of 12 independent trajectories.

The Voronoi cell volumes and cell-face counts (neighbours) represent the local packing environments of Ca particles [Supplementary Figure 2, panels (a) and (b), and Supplementary Figure 3, panels (a) and (b)]. These properties are (of course) strongly correlated to the density, which is equal for all configurations by construction. We note the asymmetry to the Voronoi cell volume distributions, which is indicative of a common global structural heterogeneity.

We also use the distribution of ring sizes to probe the structural similarity on longer length scales [Supplementary Figure 2, panel (c), and Supplementary Figure 3, panel (c)]. The graph was constructed by assigning periodic edges to particles with an interatomic separation $<$ 5.20 Å, corresponding to the minimum after the first peak in $g_{\mathrm{Ca}}(r)$. A ring is defined as a cycle which is not the sum of two strictly smaller cycles. We also compute the distribution of strong ring sizes [Supplementary Figure 2, panel (d), and Supplementary Figure 3, panel (d)], where a strong ring is defined as a cycle which is not the sum of any number of strictly smaller cycles.

As a final check, we computed the three-body correlation functions, $g_3(r,\theta)$, which are proportional to the probability of finding a second neighbour, C, from a central atom, B, at a distance $r_{\mathrm{B}C}$ and angle $\theta_{\mathrm{A}BC}$, where atom A is the nearest neighbour of B. We find that the resulting correlation functions [Supplementary Figure 4] are almost identical for the HRMC and LJG-based calcium distributions, lending further weight to the quality of the LJG description of

effective Ca$\cdots$Ca interactions.

Supplementary References

[53] Clark, S. M. *et al.* The nano- and meso-scale structure of amorphous calcium carbonate. *Sci. Rep.* **12**, 6870 (2022).

[54] Sukhomlinov, S. V. & Müser, M. H. A mixed radial, angular, three-body distribution function as a tool for local structure characterization: Application to single-component structures. *J. Chem. Phys.* **152**, 194502 (2020).

[55] Ramasubramani, V. *et al.* freud: A software suite for high throughput analysis of particle simulation data. *Comput. Phys. Commun.* **254**, 107275 (2020).

[56] Zoubritzky, L. Liozou/periodicgraphs.jl: Extend graphs.jl to manipulate periodic graphs. URL https://github.com/Liozou/PeriodicGraphs.jl.git.
